# Supplementary figures and images for: Affective touch reduces histamine evoked itch experience
Source: PLoS One. 2025 Apr 22;20(4):e0319006. doi: 10.1371/journal.pone.0319006 (PMC12013876; doi:10.1371/journal.pone.0319006)

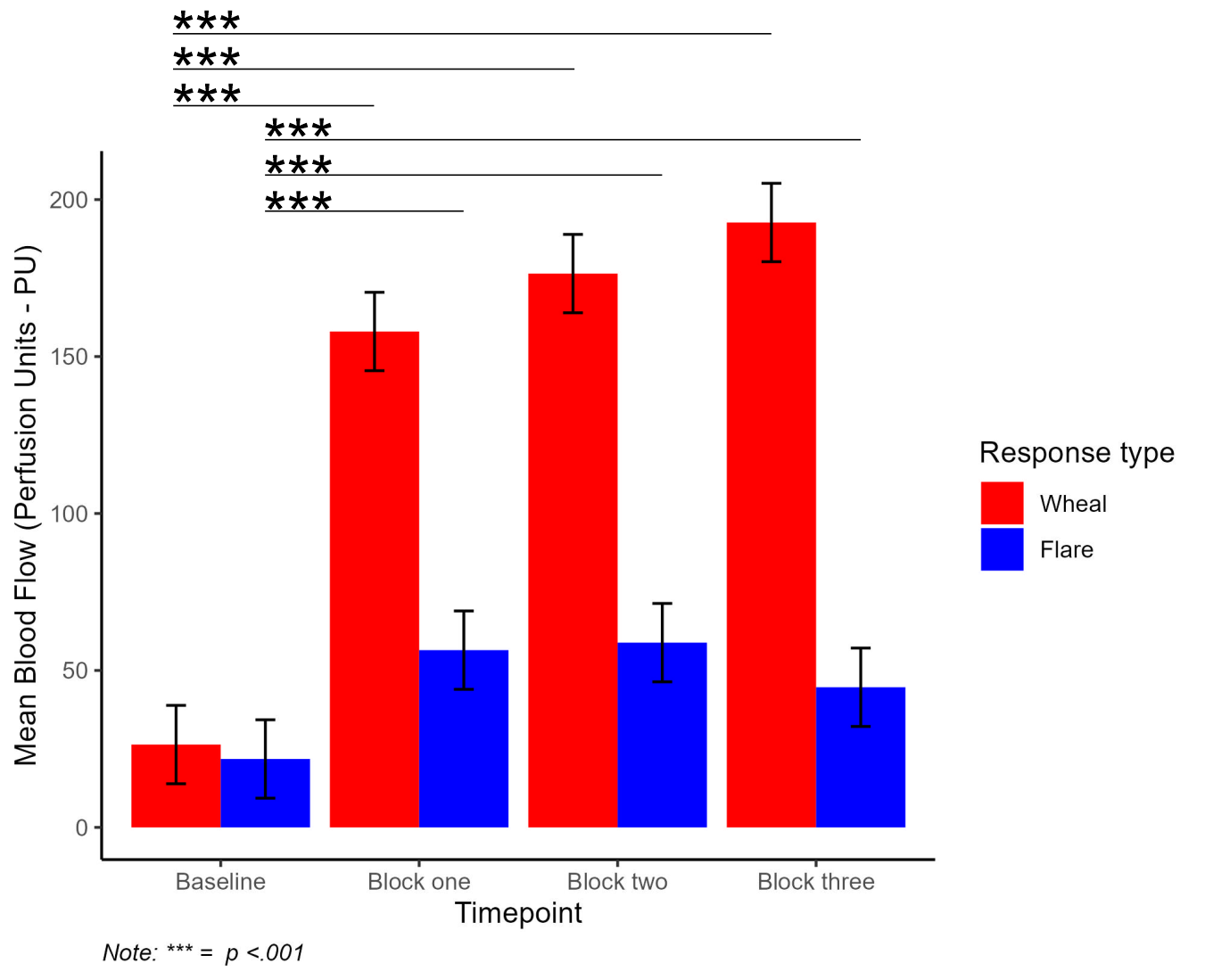

Supplement: S1 Fig — Compared to baseline, there was a significant difference in the mean wheal and flare response after iontophoresis compared to baseline. (TIF) [file pone.0319006.s001.tif]

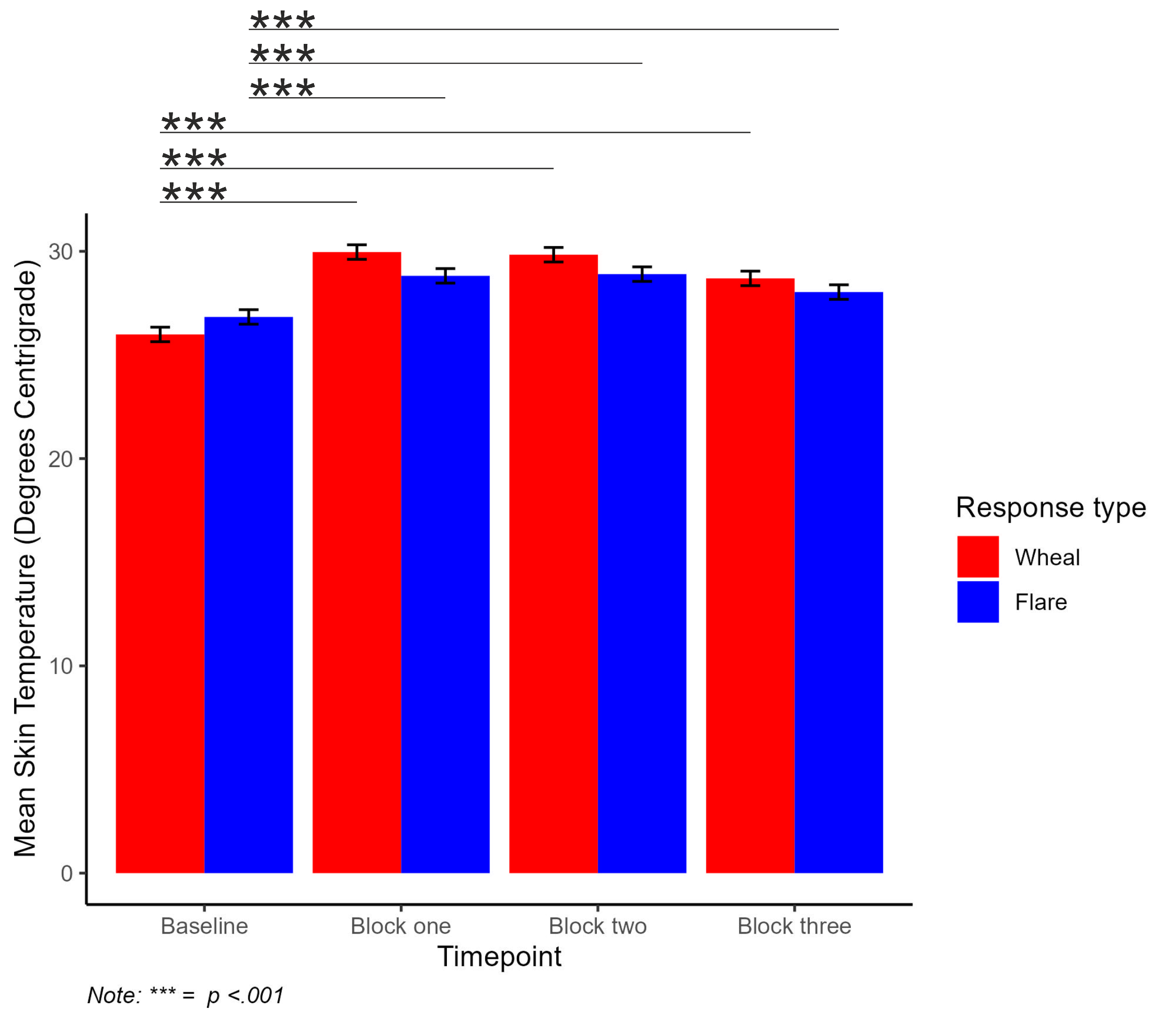

Supplement: S2 Fig — Compared to baseline, there was a significant difference in the mean skin temperature wheal and flare response after iontophoresis compared to baseline. (TIF) [file pone.0319006.s002.tif]
