# Supplementary material for: Affective touch reduces histamine evoked itch experience
Source: PLoS One. 2025 Apr 22;20(4):e0319006. doi: 10.1371/journal.pone.0319006 (PMC12013876; doi:10.1371/journal.pone.0319006)
Supplement: Attachment — Submitted filename: Response to Reviewers.docx [file pone.0319006.s004.docx]

Hasan Ali

University of Liverpool

Elanor Rathbone Building

Liverpool, UK, L69 7AZ

[Hasan.Ali@Liverpool.ac.uk](mailto:Hasan.Ali@Liverpool.ac.uk)

December 2024

Dear Mr Santosh Kimar Mishra,

We would like to thank the reviewers for their comments and the invitation to revise our manuscript titled “Affective touch reduces histamine evoked itch experience” (manuscript number: PONE-D-24-28847). We have addressed each comment below and incorporated changes to the revised manuscript as tracked changes. We feel that the manuscript has now improved significantly and hope this will be acceptable for publication in PlosOne.

Reviewer #1: Each block consisted of 6 trials across 3 conditions, and each subject completed 3 blocks. How was the data analyzed to generate a single data point for each condition per subject?

We thank the reviewer for this comment and agree this needed clarifying in the manuscript. We have added the following to the “Data processing and analysis” section of the manuscript:

“For each participant, average scores for itch severity and itch pleasantness were calculated for each of the 3 conditions, for each of the 3 blocks of the experiment (i.e. average of two trials per condition for each block), using the ‘tidyverse’ package in R (version 2.0.0).”

Reviewer #1: Histamine was applied to the same arm in blocks 1 and 3 within a short time span. It's likely that the first application influenced the response to the third application.

We thank the reviewer for this insight. We have added the following to the discussion to address this point:

“Another potential limitation of the current study is that histamine was applied to the same arm in blocks one and three, meaning that the first application may influence the response to the third application (e.g. via an additive effect of histamine). However, despite there being a main effect of block for both itch severity and brushing pleasantness, there was no block-by-condition interaction, as such this does not impact the interpretation of the results.”

Reviewer #1: It seems unlikely that itch intensity remained stable across 18 trials.

We agree with the reviewer, however we feel that the study design accounted for this with randomisation of trial type within each block. We have added the following to the discussion as a limitation.

“In addition, it is possible that itch intensity was not entirely stable across trials. However, given the randomisation of order of trial type across blocks we do not feel that this impacts the interpretation of the findings.”

Reviewer #1: The study design assumes that the inhibitory effect of affective touch does not carry over to subsequent trials. Is this assumption valid?

Given trials were randomised across blocks, the between conditions differences in effect suggests that inhibitory effects do not carry over to other trial types. Or if they do then actual differences between conditions are in fact even greater.

Reviewer # 1: The complexity of the study design raises questions about its rationale. A schematic of the experimental timeline would be helpful.

We politely disagree with the reviewer about questions over its rationale – especially given that the design of the study has circumvented each of the potential limitations from the 3 previous comments. However, we agree that a schematic would be helpful, and so this has now been added as Fig 2 in the revised submission.

Reviewer #1: There is inconsistency between the methods and abstract regarding the locations of histamine iontophoresis and brushing. Was the experiment conducted consistently?

We must apologise for our error here. The inconsistency between the methods and abstract has now been corrected.

Reviewer #1: Each brushing trial lasted 20 seconds, followed by an itch rating. Were all trial durations consistent?

Each brushing trial was consistently 20 seconds long. This was kept to 20 seconds by a visual metronome visible to the researcher which also guided the speed at which the brushing was done. During the rating of itch severity and brushing pleasantness, participants had as much time as they required but were instructed to make ratings as quickly as possible. The manuscript has now been updated to improve the clarity of the duration of trials.

“Each brushing trial lasted 20 seconds, and trial types/conditions were randomised. A visual metronome visible to the researcher was used to guide the velocity to which the brushing and tapping was performed. This metronome also guided the timing of trials.”

Reviewer #1: Skin temperature and perfusion results were not reported.

We apologise for the confusion here. The skin temperature and perfusion results were conducted to ensure that the participants in our sample had the typical response to histamine iontophoresis. We have added the following to the methods section (below), and added the results to supplementary materials

“Results from the laser doppler skin perfusion and skin temperature monitors showed that participants demonstrated a standard physiological response to histamine (see supplementary materials for full an explanation of data analysis and results).”

Reviewer #1: Individual data points should be displayed in the graphs.

The graphs have now been amended so that individual data points are displayed in the graphs.

Reviewer #1: Why were the majority of subjects female?

This was not intentional but was an effect of opportunistic testing from primarily psychology student sample at the University of Liverpool. We have added the following acknowledgement of this limitation in the discussion:

“It is also noteworthy that the majority of participants in the current sample were female, which may reduce generalisability of the results. Indeed there is evidence to suggest that females perceive affective touch to be more pleasant than males [83]. However, the results from this study showed that itch severity was reduced independently of the pleasantness of touch. Future studies may wish to recruit equal numbers of males and females to investigate any possible sex differences.

Reviewer #2: This is an important study that indicates that affective touch can alleviate histaminergic itch in human subjects. Overall the experiments are well designed, the analyses is technically sound, and the knowledge gained can have clinical implications.

We would like to thank the reviewer for their kind comments and are hoping that the results from this study does have clinical implications.

Yours sincerely,

Hasan Ali
